# Diagnosis and Treatments of Limb Lymphedema: Review

**DOI:** 10.3400/avd.ra.24-00011

**Published:** 2024-03-13

**Authors:** Shinya Kitayama

**Affiliations:** 1 Department of Orthopedic Surgery, Yokohama City University Hospital, Yokohama, Kanagawa, Japan

**Keywords:** lymphedema, diagnosis, treatment

## Abstract

Lymphedema is caused by dysfunction of the lymphatic system. It is divided into primary edema with no apparent cause and secondary edema with an exogenous cause. The main symptoms are edema and heaviness, skin changes such as skin hardening, lymphocysts, lymphorrhoea, papillomas, and recurrent cellulitis. They are often irreversible and progressive, thus greatly reducing quality of life of the patients. Diagnosis is made by image examinations that can evaluate lymphatic flow and functions such as lymphoscintigraphy and indocyanine green fluorescence lymphangiography. Linear pattern and dermal backflow are the main findings. Conservative treatment consists of four components: compression therapy with elastic garments, exercise therapy, manual lymphatic drainage, and skin care, which is called complex physical therapy (CPT). Although CPT has become the gold standard of treatment, with evidence of efficacy reported in terms of volume reduction, maintenance, and prevention of cellulitis, it is a symptomatic treatment and does not improve impaired lymphatic flow. On the other hand, surgical treatment, such as lymphaticovenous anastomosis and vascularized lymph node transplantation, can create new lymphatic flow and improve lymphatic dysfunctions. Although these techniques are expected to be effective in volume reduction, cellulitis prevention, and improving quality of life, there is a need for more studies with a higher level of evidence in the future. In Japan, lymphedema is treated with a combination of conservative and surgical therapies, but lymphedema is intractable and few cases are completely cured. Therefore, how to improve the outcome of treatment is an important issue to be addressed in the future. (This is a translation of Jpn J Vasc Surg 2023; 32: 141–146.)

## Definition of Lymphedema

Lymphedema is edema caused by the delayed return of tissue fluid due to dysfunction of the lymphatic system. Lymphedema is divided into primary and secondary according to the underlying cause. Primary lymphedema has no apparent cause; thus, congenital malformation of the lymphatic system is thought to be the primary cause. While some familial cases with genetic abnormalities are found, most cases are isolated. Primary lymphedema is subdivided into congenital (<1 year of age), early onset (1–35 years of age), and late onset (≥35 years of age) according to the time of onset.^1)^

On the contrary, secondary lymphedema is attributed to exogenous causes that invasively impair the lymphatic system. Typical invasive cases include lymph node dissection, chemotherapy (particularly with taxanes), radiotherapy, trauma, chronic infection, inflammation, and filariasis. In developed countries, including Japan, most cases involve the first three of these invasive causes and thus occur as sequelae to cancer treatment. In the vast majority of cases of secondary lymphedema of the upper limbs, the underlying disease is breast cancer; thus, this condition is called breast cancer treatment-related lymphedema. Gynecological cancers, such as uterine cancer and ovarian cancer, commonly underlie secondary lymphedema of the lower limbs. Accordingly, it has been reported that secondary lymphedema is more common in women, with a male-to-female ratio of approximately 1:9. Primary lymphedema is also more common among women with a male-to-female ratio of 1:3–9. These data indicate that most lymphedema patients are female.

Primary lymphedema is relatively rare, and the estimated number of patients in Japan is approximately 3500–4000.^2)^ Secondary lymphedema accounts for most instances of lymphedema, and the total number of patients in Japan is estimated to be approximately 100000; however, the exact figure is unknown.

## Diagnosis of Lymphedema

### Primary symptoms and physical findings

The primary symptoms of lymphedema include edema and associated heaviness, lassitude, and skin changes such as skin and subcutaneous tissue hardening, lymphocysts, lymphatic fistulas, papillomas, and recurrent cellulitis. Edemas are characterized by a transition with progression from initial pitting edemas to nonpitting edemas, without changes in skin color or pain. Skin changes appear as the disease progresses, and in patients with bilateral edema in particular, they occur in the pubic region and lower abdomen, reducing their quality of life (QOL). When lymphatic congestion occurs and intralymphatic pressure increases, lymph duct degeneration and hardening progress in an irreversible manner, making symptoms often progressive. Furthermore, patients with lymphedema are prone to developing cellulitis recurrently because they have abnormalities in local immunity.^3^^–^^5)^ Once inflammation occurs, severe inflammation further reduces lymphatic function and increases the likelihood of recurrent inflammation; due care should be paid to such a typical vicious circle.

## Tests

### Lymphoscintigraphy/single-photon emission computed tomography-computed tomography (SPECT-CT) lymphoscintigraphy

This is a test to obtain images of the lymphatic system using radioisotope-labeled colloid as a contrast medium, which is injected intracutaneously–subcutaneously and taken up by the lymphatic system. Technetium-99m-labeled human albumin is often used as the contrast medium.

The subcutaneous contrast medium physically enters the lymph ducts, where it is distributed based on the subject’s lymph functioning; therefore, lymph functioning can be panoramically evaluated. Because the contrast medium taken up in the body migrates in the lymphatic system proximally over time, different images are obtained depending on the length of time between the injection and image acquisition. While there is no consensus on when and how many images should be acquired, images are generally acquired twice: in the early phase at approximately 30 minutes after injection and in the late phase at 90–120 minutes after injection.

**Figure 1:**
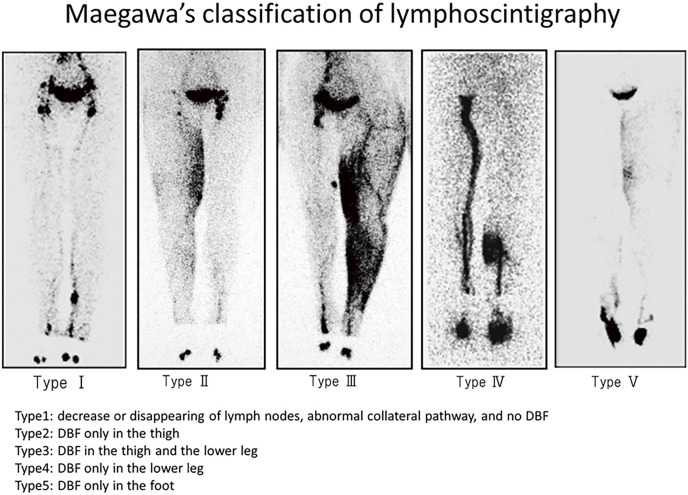
Fig. 1 Maegawa’s classification of lymphoscintigraphy. The pattern of LP and DBF changes as the lymphatic vessels degenerate progres (modified and quoted from reference 6). LP: linear pattern; DBF: dermal back flow

Lymph ducts present a linear pattern (LP), and lymph nodes present a granular pattern of contrast enhancement. When lymph flow is congested, lymphatic fluid with nowhere to go flows back to the lymphatic capillaries of the skin and is delineated in a reticulated or planar form called dermal back flow (DBF). In secondary lymphedema, the LP and DBF delineation patterns change depending on the disease stage because degeneration gradually proceeds from the proximal regions to the distal regions of the limbs. Maegawa et al. classified these lymphoscintigraphy patterns into five types, which represent the stages and severity levels of lymphedema (**Fig. 1**), and this classification is used to evaluate the state of lymphedema.^6)^

In SPECT-CT lymphoscintigraphy, tomographic images of the distribution of contrast medium are obtained using multiple gamma cameras and fused with a plain CT image. The contrast medium accumulation site is delineated as a hot spot on each tomographic image so that lymph flow and congestion can be evaluated in a three-dimensional (3D) manner.^7)^ While lymphoscintigraphy is useful for evaluating the overall picture of lymph flow, it is insufficient for evaluating the precise location of lymph ducts. On the contrary, because SPECT-CT enables the 3D evaluation and localization of lymph flow, a great deal of information can be obtained, such as whether the lymph duct on an image is superficial or deep; however, the image acquisition process is complex, and there is radiation exposure in CT, which makes it impossible to obtain multiple images in different phases, such as in lymphoscintigraphy, and images are usually only taken in the late phase.

As mentioned above, both lymphoscintigraphy and SPECT-CT lymphoscintigraphy are very useful for the diagnosis and evaluation of lymphedema; however, to obtain images, an expensive gamma camera and an imaging room with a radiation-shielding environment are needed. Therefore, these methods are available only in large-scale medical institutions, such as university hospitals, and it is difficult to use such imaging methods to screen for lymphedema on an outpatient basis at general hospitals or clinics.

### Indocyanine green fluorescence lymphangiography (ICG-FL)

**Figure 2:**
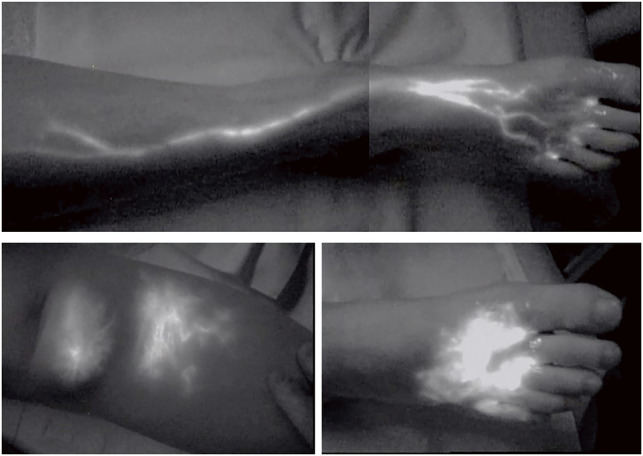
Fig. 2 ICG fluorescent lymphangiography. Above: Normal case, LP is seen from dorsal foot to lower leg, and no abnormal findings indicating congestion. Below: Patient with lymphedema, and DBF appears on the dorsal foot (right image) and around the knee (left image). ICG: indocyanine green; LP: linear pattern; DBF: dermal back flow

ICG-FL is a relatively new lymph flow testing method developed by Unno et al.^8)^ in Japan and is widely used worldwide for its effectiveness, simplicity, and low invasiveness. Using ICG as a contrast medium, lymph flow can be visualized from the body surface using an ICG characteristic of emitting fluorescence when it receives infrared rays. An infrared camera, such as the PDE neo (Hamamatsu Photonics, Co. Ltd., Hamamatsu, Japan), is needed to delineate fluorescence imaging; however, the device is not so expensive, is relatively compact and mobile, and does not require radiation control. Thus, the method can be implemented at a wide range of institutions. Findings that can be obtained are LP and DBF as per lymphoscintigraphy; however, unlike lymphoscintigraphy, real-time images can be observed directly from the body surface (**Fig. 2**). Therefore, ICG-FL is applied to not only diagnose but also determine the surgical site and postoperative evaluation. The thickness through which fluorescence emitted by ICG can pass is up to approximately 1.5 cm; hence, the inability to detect deep lymph flow is a shortcoming. Furthermore, noise occurs when there is light containing near-infrared radiation, such as light from the sun and incandescent lamps, in environments; therefore, shielding of such light is needed (LED light does not include infrared light and can thus be used).

ICG-FL can be implemented relatively easily without selecting the institution or place compared to lymphoscintigraphy and can be easily applied for the screening of and regular evaluations of lymphedema. In 2018, Shinaoka et al. conducted a questionnaire survey on the state of lymphedema imaging examinations in medical institutions in the field of orthopedic surgery in Japan; the survey results showed that ICG-FL was performed at 77% of institutions,^9)^ and at present, it is believed that ICG-FL is performed at even more institutions. Despite this, ICG-FL is still not covered by health insurance in Japan, and tests are presumably performed at patients’ own cost, using research funds, or at the expense of hospitals, which is impeding the widespread availability of ICG-FL in general medical institutions. To address this situation, Akita et al. conducted an investigator-led multicenter phase III trial to evaluate the usefulness of ICG-FL for the treatment of lymphedema and provided evidence that ICG-FL is useful for the diagnosis and treatment of lymphedema.^10)^ Under such circumstances, ICG-FL should be covered by health insurance as soon as possible for the development of lymphedema care in Japan.

## Diagnosis

As mentioned above, lymphedema can be diagnosed relatively easily based on past medical history, physical findings, and test findings. In the past, diagnosis was often made by exclusion based on medical history and physical findings; however, for accurate diagnosis, it is important to perform the abovementioned lymphatic function tests and confirm the presence of lymphatic dysfunction. Differential diagnosis includes localized edema, such as venous congestion-induced edema, disuse edema, and lipedema. In venous congestion-induced edema, the amount of tissue fluid that should return increases, resulting in an increase in preload and secondary loading on the lymphatic system. As a result, collateral pathways and DBF are observed on scintigraphy, and it is difficult to differentiate them from primary lymphedema in some cases; however, in such instances, physical findings and the presence or absence of lymph node delineation in the inguinal and pubic regions can be used as bases of differentiation.

## Treatment of Lymphedema

### Conservative treatment

Conservative treatment for lymphedema consists of the following four methods: “compression therapy with compression garments,” “exercise therapy with compression,” “manual lymphatic drainage (MLD),” and “skin care,” a combination of which is called complex physical therapy (CPT). Conservative treatment has a long history, with the simple concept of allowing congestion of lymph flow proximally, and it is still the gold standard for the treatment of lymphedema.

As compression garments, stockings and bandages are used. Stockings have the advantage of providing high-pressure reproducibility. Once compression measurement is done and pressure is confirmed, application of similar levels of pressure can be expected thereafter. As shortcomings, familiarity and grip strength are required to put on and take off the stockings, and some people, such as elderly individuals, find it difficult to use them. Conservative treatment using bandages is called multilayer lymphedema bandaging (MLLB). Bandages can be wrapped more or less tightly to provide a broad pressure adjustment range from low to high compression and can be worn even by an individual with poor grip strength. However, the bandage application method needs to be learned, and if the bandage comes loose, it must be reapplied as needed. Furthermore, pressure reproducibility is not necessarily high. Relatively many pieces of evidence for the effectiveness of compression garments have been reported, primarily in terms of their volume-reducing effect, volume-maintaining effect, and cellulitis-prevention effect.^11^^–^^14)^ In the guidelines of the Japanese Lymphedema Society, compression garments have recommendation grades of B–C1, giving them a key role in conservative treatment.^15)^

Exercise, in addition to having external pressure applied by compression garments, applies pressure to the entire circumference of the lymph ducts pressed internally by muscle contraction, allowing for easy and effective lymph flow. This is called exercise therapy with compression. Because muscles contract and relax in turn during exercise, intermittent pumping actions are induced, producing a higher effect than a simple application of compression. Several reports have shown that it is particularly effective for volume reduction of the upper limbs and function improvement of the upper and lower limbs,^16^^–^^19)^ and in the abovementioned guideline, the recommendation grade for exercise therapy with compression is B–C1.^15)^

Unlike compression therapy, in which pressure is applied to the affected whole limbs, MLD is a method of draining lymphatic fluid by massaging the skin surface. In MLD, the order of and technique for drainage are defined; these have been developed and traditionally passed down based on the anatomical characteristics of the lymphatic system. MLD has a long history, and there are many reports on the effects of MLD; however, there is a paucity of reports that clearly demonstrate its effectiveness, and the situation remains that MLD is likely to be effective.^20^^–^^23)^ In the guidelines, the recommendation grade is C1–C2.^15)^

In CPT, treatment is administered in two stages; initially, strong pressure is applied to sufficiently eliminate edema fluid, and then, compression stockings are adjusted to fit the affected limbs that have become sufficiently thin. These two stages are referred to as the intensive volume-reduction phase and maintenance phase, respectively. In the intensive volume-reduction phase, MLLB and high-pressure stockings are used, and the fluid is removed by changing the garment frequently in accordance with circumferential changes until the circumference reaches a plateau. Compression stockings for maintenance are adjusted according to the final circumference; however, if edema fluid becomes congested again due to the patient’s living environment or symptoms, the treatment can be repeated from the intensive volume-reduction phase.

### Surgical treatment

**Figure 3:**
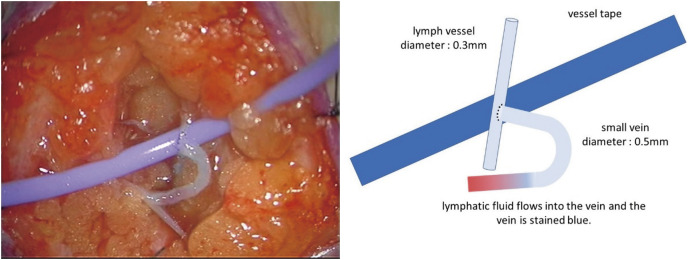
Fig. 3 Photograph and schema of lymphaticovenous anastomosis. Lymphatic fluid flows into the vein through the anastomosis.

Two typical surgical procedures currently used are lymphovenous anastomosis (LVA) and vascularized lymph node transplantation (VLNT). Compared to conservative treatment, which is a symptomatic treatment, both procedures aim to improve lymphatic dysfunction, which is the root cause of lymphedema. In LVA, lymph ducts are anastomosed to venules to drain congested lymphatic fluid into veins (**Fig. 3**). Intralymphatic pressure when lymphedema occurs has been found to be higher than the pressure within venules, and drainage and reduced intralymphatic pressure can be expected to result from anastomosis. As the method of anastomosis, end-to-end or side-to-end anastomosis is commonly selected. VLNT aims to reconstruct lymph flow by transplanting vascularized tissue containing lymph nodes. The inguinal, thoracic, and cervical areas are selected to obtain the donor tissue. As an advantage, completely new healthy tissue can be introduced into the affected area; however, the donor tissue has to be sacrificed, and there are complications, such as lymphedema onset at the site of harvesting. Therefore, it is primarily performed for severe patients for whom other treatments have been ineffective. Both LVA and VLNT require microsurgical skills to anastomose vascular channels of smaller than 1 mm.

Surgical treatment for lymphedema is a relatively new procedure, and reports of the outcomes have increased in recent years. Most reports are on case-series studies, and the recommendation grade in the guidelines is C2 as guidelines require high-quality studies; however, there are many reports showing the volume-reducing effect, cellulitis-prevention effect, and QOL improvement, and LVA and VLNT are expected to be effective.^24^^–^^26)^

Other surgical treatments include liposuction. The physical removal of subcutaneous tissue can be expected to reduce the circumference reliably and substantially. On the contrary, it involves extensive invasion of tissue, including residual lymph ducts; therefore, lifelong compression therapy is required after surgery. If postoperative compression therapy is not performed properly, the severity of lymphedema can increase compared to before surgery. Therefore, various methods are being devised, such as combination treatment with other function-improving surgeries, such as LVA.

### Our treatment policy

In lymphedema, it is difficult to assess the outcomes of individual treatments because surgical treatment is performed in addition to various conservative treatments. We have treated lymphedema for 20 years and performed LVA since the dawn of surgical treatment for functional improvement. In conservative treatment, we use stockings and measure the compression strength to apply the pressure suitable for the severity. Conservative treatment is performed until volume reduction reaches a plateau, and LVA is additionally used upon patients’ request. We evaluate the circumference, volume, and anastomosis patency regularly and change the conservative treatment or perform LVA again according to symptoms, with the ultimate aim of improving QOL by reducing or ceasing usage of compression garments. Based on our experience, volume reduction as a result of conservative treatment was approximately 11%, with an additional reduction of approximately 2% from subsequent LVA.^26)^ The anastomosis patency rate was approximately 75% 1 year after surgery and 35% 2 years after surgery.^27)^ To date, patency has been observed up to 8 years after surgery. Complex treatment combining conservative treatment and LVA not only improves the circumference but also reduces the incidence of cellulitis, and for the upper limbs in particular, conservative treatment can be reduced or ceased in many cases. While a certain degree of stability is often obtained, treatment of lymphedema in the lower limbs remains problematic because conservative treatment can be rarely eased and there are progressive refractory cases.

## Disclosure Statement

I have no conflicts of interest with other companies related to this paper.

## Additional Remark

This paper was presented at the 34th educational seminar of the 50th Annual Meeting of the Japanese Society for Vascular Surgery (2022, Kitakyushu, Fukuoka prefecture).
